# SARS-CoV-2 Infection Associated with HHV-6A Reactivation and an Inhibitory KIR2DL2/HLA-C1 Immunogenetic Profile

**DOI:** 10.3390/microorganisms14010235

**Published:** 2026-01-20

**Authors:** Sabrina Rizzo, Matteo Ferraresi, Giovanni Strazzabosco, Marcello Baroni, Juana Maria Sanz, Angelina Passaro, Daria Bortolotti, Roberta Rizzo, Giovanna Schiuma

**Affiliations:** 1 Department of Environmental and Prevention Sciences, University of Ferrara, 44121 Ferrara, Italy; sabrina.rizzo@unife.it (S.R.); matteo.ferraresi@unife.it (M.F.); giovanni.strazzabosco@unife.it (G.S.); giovanna.schiuma@unife.it (G.S.); 2 Department of Life Sciences and Biotechnology (SVEB), University of Ferrara, 44121 Ferrara, Italy; marcello.baroni@unife.it; 3 Department of Chemical, Pharmaceutical and Agricultural Sciences, University of Ferrara, 44121 Ferrara, Italy; juana.sanz@unife.it; 4 Department of Translational Medicine, University of Ferrara, 44121 Ferrara, Italy; angelina.passaro@unife.it

**Keywords:** KIR2DL2, HLA-C1, NK cells, HHV-6A, co-infection, COVID-19, comorbidities

## Abstract

Natural killer (NK) cells are central to antiviral immunity through a balance of activating and inhibitory receptors, including killer immunoglobulin-like receptors (KIRs). We have previously observed that an increased frequency of the inhibitory receptor KIR2DL2 and its ligand HLA-C1 is associated with heightened susceptibility to human herpesvirus (HHV) infection, supporting a role for KIR-mediated NK cell regulation in host–virus interactions. We investigated whether the co-infection of SARS-CoV-2 and human herpesvirus 6 (HHV-6) might be connected to the expression of KIR2DL2/HLA-C1. We analyzed 110 SARS-CoV-2-positive subjects and 109 SARS-CoV-2-negative subjects for the KIR2DL2 and HLA-C1 genotype and for HHV-6A/B reactivation in plasma samples. SARS-CoV-2-positive subjects showed a significantly higher frequency of the KIR2DL2/HLA-C1 haplotype and increased reactivation of HHV-6A. Among deceased and comorbid patients, the co-occurrence of the KIR2DL2/HLA-C1 haplotype and HHV-6A DNAemia was more frequent, particularly in those with cardiovascular disorders. These findings suggest that the KIR2DL2/HLA-C1 haplotype might promote NK cell inhibition, facilitating HHV-6A persistence and contributing to immune dysregulation during SARS-CoV-2 infection. The combined presence of KIR2DL2/HLA-C1 and HHV-6A may, therefore, represent a molecular signature of COVID-19 outcomes.

## 1. Introduction

In December 2019, severe acute respiratory syndrome coronavirus 2 (SARS-CoV-2) was isolated in Wuhan, China, and identified as an emerging pathogen [1]. SARS-CoV-2 is a betacoronavirus with a positive-sense single-stranded RNA genome that binds the angiotensin-converting enzyme 2 (ACE2) receptor via its spike protein [2,3,4]. The virus spreads efficiently via the respiratory route and rapidly caused a global pandemic.

SARS-CoV-2 infection causes coronavirus disease 19 (COVID-19), a respiratory syndrome ranging from mild illness to severe pneumonia and acute respiratory distress syndrome (ARDS) [5,6]. Severe disease is frequently associated with marked immune dysregulation affecting both innate and adaptive responses, with alterations that may compromise early antiviral control and amplify inflammatory injury [7]. Among innate immune abnormalities described in severe COVID-19, decreased monocyte counts [8,9], reduced neutrophil adhesion [10], and profound perturbations of natural killer (NK) cell number and function [11,12] have been reported. In particular, NK cell anergy/exhaustion has emerged as a recurring feature in hospitalized patients, and mechanistic work by Bortolotti et al. showed that the spike protein can contribute to an exhausted NK cell phenotype characterized by reduced interferon-γ (IFN-γ) secretion and impaired cytotoxicity [13]. Because NK cells are crucial for the rapid clearance of virus-infected cells, such defects may influence susceptibility, viral persistence, and downstream inflammatory trajectories.

Within the immune system, NK cells play a pivotal role in antiviral defense [14]. Their effector functions depend on a balance of activating and inhibitory receptors expressed on the cell surface, including killer immunoglobulin-like receptors (KIRs) that bind human leukocyte antigen class I (HLA-I) molecules [15,16]. KIR/HLA interactions shape NK cell “education” and tune activation thresholds, thereby influencing the efficiency and timing of NK cell responses during viral infection. Notably, inhibitory KIR/HLA combinations can dampen cytotoxic activity and cytokine production [17], potentially reducing early containment of viral replication and creating a permissive setting for viral persistence [18,19] or reactivation [20].

Among inhibitory KIRs, KIR2DL2 and its ligand HLA-C1 have been associated with higher susceptibility to viral infections, including herpesviruses (HHVs), because their interaction limits NK cell activation [21,22]. This pathway may be exploited by HHVs to evade immune surveillance. Human herpesvirus 6 (HHV-6) is a ubiquitous β-herpesvirus that establishes latency after primary infection and can reactivate under conditions of immune perturbation. Two species are recognized, HHV-6A and HHV-6B, which differ in epidemiology, tissue tropism, and immunobiological properties [23,24]. Several studies have reported that both HHV-6A and HHV-6B can increase HLA-C1 expression, thereby engaging KIR2DL2 and inhibiting NK cell responses, which may facilitate viral persistence. Clinically, HHV-6B typically causes roseola infantum in healthy children [25], whereas HHV-6A has been implicated more often in chronic and immune-mediated conditions, including myalgic encephalomyelitis/chronic fatigue syndrome [26,27]. In addition, in neuroinflammatory disorders such as multiple sclerosis, KIR2DL2-expressing NK cells show reduced activation against HHV-6—predominantly HHV-6A—supporting a functional link between inhibitory KIR/HLA profiles and impaired control of this virus. Collectively, this body of evidence highlights the importance of KIR/HLA interactions in antiviral immunity and their involvement in human disease.

NK cell function is also fundamental in counteracting SARS-CoV-2 infection, and NK cell dysfunction in COVID-19 may impair antiviral control and increase vulnerability to co-infections and herpesvirus reactivation [28]. HHVs typically alternate between acute and latent phases, with latency being tightly controlled by NK cells and other immune effectors [29,30]. Accordingly, HHV-6 reactivation or co-infection has been reported with notable frequency in hospitalized COVID-19 patients [13,14], and it has been proposed that herpesvirus reactivation may exacerbate acute disease and contribute to longer-term sequelae, including long COVID [31,32,33]. In this framework, herpetic reactivation may represent a risk factor for COVID-19 complications, together with established clinical determinants such as age, sex, and comorbidities [34,35]. However, the immunogenetic factors that predispose to herpesvirus reactivation in the context of SARS-CoV-2-associated NK cell dysregulation remain insufficiently characterized.

In the context of NK cell dysregulation, the presence of the inhibitory KIR2DL2/HLA-C1 pairing may plausibly reduce control of viral infections during SARS-CoV-2 infection [36,37]. Importantly, the distribution of KIR and HLA genotypes varies among populations, and such immunogenetic diversity can influence susceptibility to viral infections and contribute to heterogeneity in clinical outcomes [38,39,40]. Thus, integrating immunogenetic information with virological readouts could help identify host–virus signatures associated with infection susceptibility, viral reactivation, and adverse outcomes.

Here, we evaluated KIR2DL2/HLA-C1 haplotype distribution and HHV-6A/B reactivation in SARS-CoV-2-positive patients and examined their relationships with comorbidities and adverse outcomes. By combining KIR/HLA immunogenetics with subtype-specific HHV-6 detection in plasma, we aimed to define whether an inhibitory KIR2DL2/HLA-C1 profile is enriched in SARS-CoV-2 infection and whether it co-segregates with HHV-6A or HHV-6B DNAemia, thereby providing insight into NK cell-mediated vulnerability pathways relevant to COVID-19.

## 2. Materials and Methods

### 2.1. Study Populations and Sample Collection

The analysis was conducted on blood samples obtained from 110 SARS-CoV-2-positive subjects and 109 SARS-CoV-2-negative subjects. None of the enrolled subjects received vaccination against SARS-CoV-2 at the time of enrollment and presented no prior SARS-CoV-2 infection during serological analysis. All subjects were enrolled between May and December 2020 at the Internal Medicine Unit of the Sant’Anna University Hospital in Ferrara. The SARS-CoV-2-infected subjects presented the B.1 lineage with the D614G spike mutation.

This study was approved by our hospital’s ethics committee (Number: 540/2020/Oss/AOUFe—20 May 2020). From each subject, blood was collected for genomic DNA extraction.

### 2.2. KIR2DL2 and HLA-C1 Evaluation

Genomic DNA was extracted from whole blood using the QIAamp DNA Blood Mini Kit (QIAGEN, Hilden, Germany) and quantified by spectrophotometry at 260/280 nm. KIR2DL2 and HLA-C1 alleles were revealed by Polymerase Chain Reaction (PCR) using specific primer pairs: KIR2DL2-F 5′-CCA TGA TGG GGT CTC CAA A-3′; KIR2DL2-R 5′-GCC CTG CAG AGA ACC TAC A-3′; HLA-C1-F 5′-CGC CGC GAG TCC RAG AGG-3′; HLA-C1-R 5′-GTT GTA GTA GCC GCG CAG G-3′. Amplicons were resolved on 1% agarose gels (KIR2DL2, ~1800 bp; HLA-C1, 139 bp).

KIR2DL2 and HLA-C1 PCR was performed using a reaction mix composed of 1× Platinum II PCR Buffer(ThermoFisher Scientific, Waltham, MA, USA), 0.2 mM dNTP mix (ThermoFisher Scientific, MA, USA), 0.2 µM specific forward (F) and reverse (R) primers, and 0.04 U/µL Platinum II Taq Hot-Start DNA Polymerase (ThermoFisher Scientific, MA, USA). KIR2DL2 amplification protocol was 95 °C for 5 min (×1); 97 °C for 20 s, 62 °C for 45 s; and 72 °C for 1 min 30 s (×5), followed by 95 °C for 20 s and 60 °C for 45 s (×25) [41]. HLA-C1 amplification protocol was 94 °C for 2 min (×1); 94 °C for 15 s, 60 °C for 15 s, and 68 °C for 15 s (×35) [42].

### 2.3. HHV-6 Detection and Species Identification

Quantitative real-time PCR (qPCR) for the U94 gene was used to detect HHV-6 DNA as previously described in plasma samples [43,44]. This method has a sensitivity of 20 copies/mL, based on a 6-log dynamic range, calculated on a standard curve generated by amplification of a plasmid containing the targeted HHV-6 sequences. We defined HHV-6 reactivation in plasma as quantifiable HHV-6 DNA ≥ 10^2^ copies/mL. Prior clinical studies in immunocompromised cohorts (e.g., allogeneic HSCT recipients) have used thresholds of ~200 copies/mL to indicate reactivation based on assay sensitivity [45]. Human RNase P or β-actin served as housekeeping controls. Primer/probe sequences were HHV-6 U94(+) 5′-GAG CGC CCG ATA TTA AAT GGA T-3′; HHV-6 U94(−) 5′-GCT TGA GCG TAC CAC TTT GCA-3′; HHV-6 U94 probe 5′-FAM-CTG GAA TAA TAA AAC TGC CGT CCC CAC C-TAMRA-3′. In positive samples, HHV-6A versus HHV-6B was distinguished by HindIII digestion of the U31 nested-PCR product [46], followed by agarose gel electrophoresis.

### 2.4. Statistics

Analyses were performed using GraphPad Prism version 10 (GraphPad Software, San Diego, CA, USA). Differences in frequencies were assessed using the χ^2^ test or Fisher’s exact test as appropriate. Bonferroni’s correction was applied for multiple comparisons. Variables reported as mean ± SD were compared between study groups by Student’s *t*-test. Two-sided *p*-values < 0.05 were considered statistically significant.

## 3. Results

### 3.1. Characteristics of the Study Population

Demographic and clinical characteristics are summarized in Table 1. The two populations were age-matched.; however, the SARS-CoV-2-negative subjects included a higher proportion of females (Table 2, *p* < 0.05, Fisher’s exact test).

### 3.2. KIR2DL2/HLA-C1 Frequency

**Figure 1:**
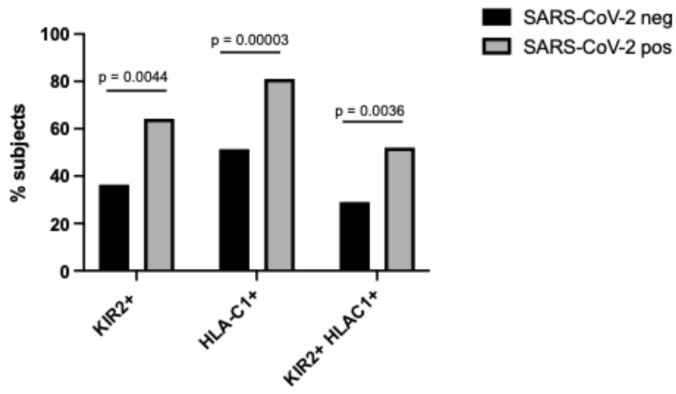
Frequency of KIR2DL2 (KIR2+), HLA-C1 (HLA-C1+) and the KIR2DL2/HLA-C1 haplotype (KIR2+ HLA-C1+) in SARS-CoV-2-positive and -negative subjects.

The two cohorts were evaluated for the frequency of the inhibitory KIR2DL2 receptor and its ligand, HLA-C1. SARS-CoV-2-positive subjects showed a higher frequency of KIR2DL2 and HLA-C1 compared with SARS-CoV-2-negative subjects (Figure 1, KIR2DL2 64.2% vs. 36.4%, respectively, *p* = 0.0044, Chi-square test; HLA-C1 81% vs. 51.4%, *p* = 0.00003, Chi-square test).

The KIR2DL2/HLA-C1 haplotype was more frequent in SARS-CoV-2-positive subjects in comparison with SARS-CoV-2-negative subjects (Figure 1, 52% vs. 29%, *p* = 0.0036, Chi-square test).

### 3.3. HHV-6A and HHV-6B Reactivation

**Figure 2:**
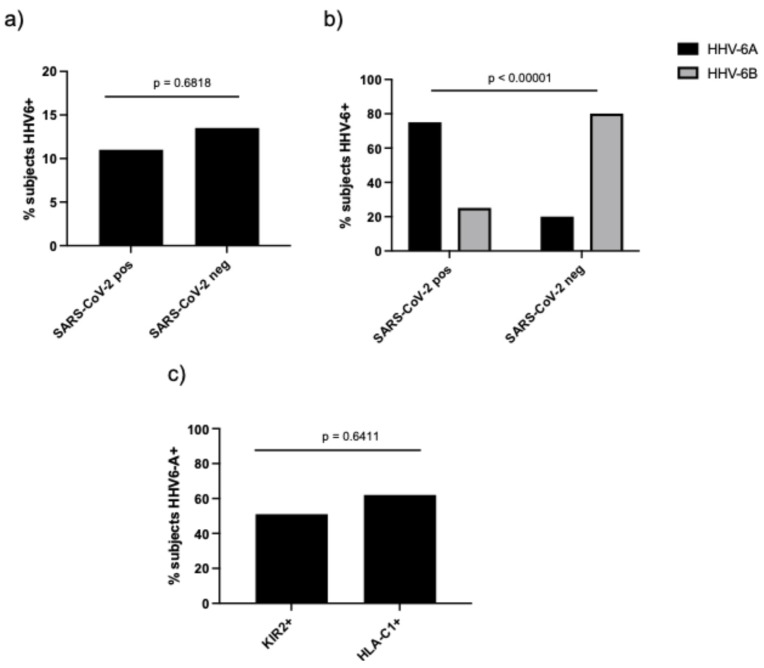
Frequency of plasma samples with >10^2^ copies/mL: (**a**) HHV-6 DNA and (**b**) HHV-6A and HHV-6B DNA in SARS-CoV-2-positive and -negative subjects; KIR2DL2 (KIR2+), and HLA-C1 (HLA-C1+)distribution in HHV-6A-positive subjects (**c**).

Overall, the percentage of plasma samples with HHV-6 DNA > 10^2^ copies/mL, suggestive of viral reactivation, did not differ significantly between SARS-CoV-2-positive and -negative samples (Figure 2a, 11% vs. 13.5%, *p* = 0.6818, Fisher’s exact test).

When we considered HHV-6 typing, the percentage of plasma samples positive for HHV-6A DNA was significantly higher in SARS-CoV-2-positive subjects (Figure 2b, 75% vs. 20%, *p* < 0.00001, Fisher’s exact test), while HHV-6B reactivation was over-represented in SARS-CoV-2-negative subjects (Figure 2b, 80% vs. 25%).

When we considered SARS-CoV-2-positive subjects found with HHV-6A DNA, we observed that more than 50% were positive for KIR2DL2 and HLA-C1 (Figure 2c, 51% and 62%, respectively, *p* = 0.6144, Fisher’s exact test), suggesting a possible enhanced susceptibility to both SARS-CoV-2 and HHV-6A [47].

### 3.4. Comorbidities in Relation to KIR2DL2/HLA-C1 and HHV-6

SARS-CoV-2-positive patients were evaluated for comorbidities, which might influence the infection susceptibility status. In Table 2, the comorbidities are reported.

Among SARS-CoV-2-positive subjects, cardiovascular disorders were the most frequent (Table 2, 55%), with hypertension present in more than half of the cases (Table 2, 51%). Chronic obstructive pulmonary disease (COPD) and diabetes were present in 18% and 21% of SARS-CoV-2-positive subjects, respectively. Moreover, 17% of SARS-CoV-2 subjects died during hospitalization. Conversely, no SARS-CoV-2-negative subjects showed these clinical conditions.

Then, comorbidities found in the SARS-CoV-2-positive group were correlated with KIR2DL2 and HLA-C1 presence.

**Figure 3:**
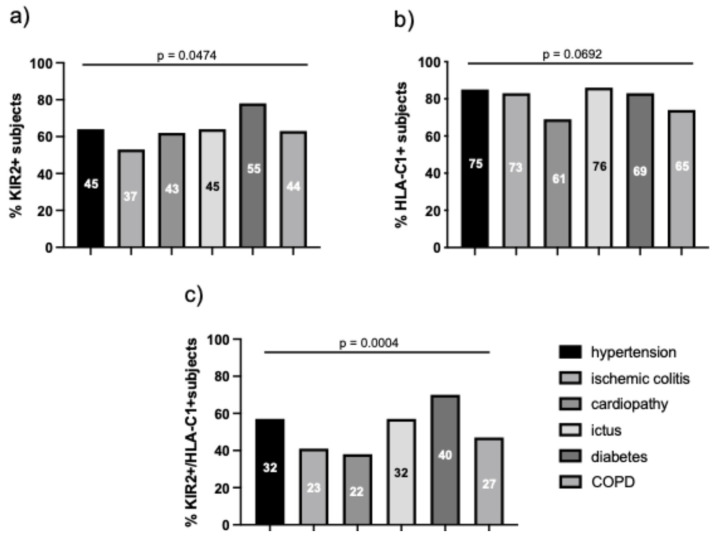
Frequency of comorbidities (hypertension, diabetes, ischemic colitis, cardiopathy, ictus, tumor, BCPO) in (**a**) KIR2DL2-positive (KIR2+), (**b**) HLA-C1-positive (HLA-C1), and (**c**) KIR2DL2/HLA-C1 haplotype (KIR2+/HLA-C1) in SARS-CoV-2-positive subjects. Numbers in histograms represent the relative number of subjects.

The correlation with KIR2DL2 and HLA-C1 frequency showed that more than 50% of subjects with comorbidities presented KIR2DL2 or HLA-C1 alleles, with diabetes more frequent in KIR2DL2-positive subjects (Figure 3a, 78%, *p* = 0.0474, Chi-square test) and hypertension, ischemic colitis, and ictus in HLA-C1-positive subjects (Figure 3b, *p* = 0.0692, Chi-square test).

The KIR2DL2/HLA-C1 haplotype was associated with diabetes (70%), ictus, and hypertension (each 57%) (Figure 3c, *p* = 0.0004, Chi-square test).

**Figure 4:**
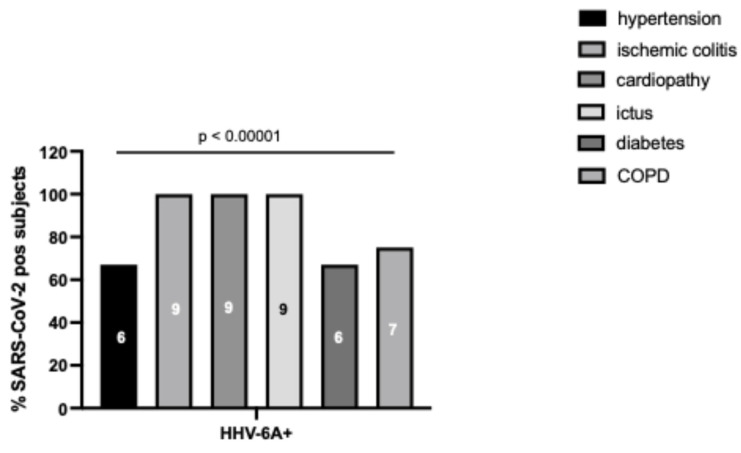
Frequency of comorbidities (hypertension, diabetes, ischemic colitis, cardiopathy, ictus, tumor, BCPO) in SARS-CoV-2-positive subjects with plasma samples with HHV-6 DNA > 10^2^ copies/mL (HHV-6+) and subjects positive for HHV-6A DNA (HHV-6A+). Numbers in histograms represent the relative number of subjects.

In SARS-CoV-2-positive subjects with plasma samples with HHV-6 DNA > 10^2^ copies/mL, the most common comorbidities were hypertension (55%) and COPD (36%) (*p* > 0.99, Chi-square test). Considering SARS-CoV-2-positive subjects positive for HHV-6A DNA, all subjects presented cardiovascular/circulatory comorbidities (ischemic colitis, heart disease, or stroke), while COPD was reported in 75% of cases (Figure 4, 100% and 75%, *p* < 0.00001, Chi-square test).

### 3.5. Mortality in Relation to KIR2DL2/HLA-C1 and HHV-6

**Figure 5:**
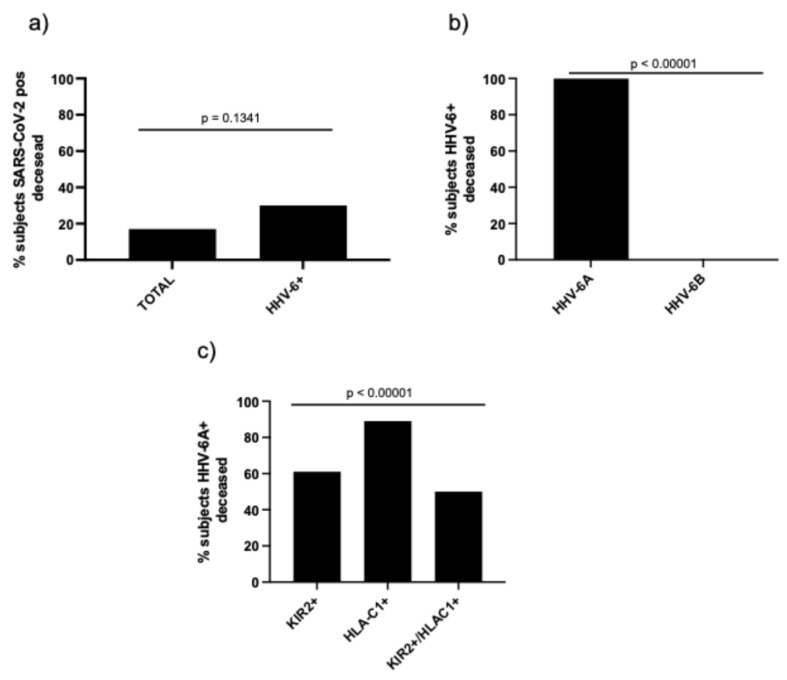
Percentage of deaths among SARS-CoV-2-positive subjects in relation to (**a**) HHV-6 DNA > 10^2^ copies/mL in plasma samples (HHV-6+); (**b**) HHV-6A or HHV-6B presence; and (**c**) frequency of KIR2DL2 (KIR2+), HLA-C1, and KIRDL2/HLA-C1 haplotype (KIR2DL2+/HLA-C1+) in deceased subjects positive for HHV-6A DNA.

Among SARS-CoV-2-positive subjects, 17% died during hospitalization (Table 2 and Figure 5a). HHV-6 DNA > 10^2^ copies/mL in plasma samples was detected in 30% of deceased subjects (Figure 5a, *p* = 0.1341; Fisher’s exact test), and all of them were HHV-6A-positive (Figure 5b, *p* < 0.00001; Fisher’s exact test).

In deceased HHV-6A-positive subjects, 61% and 89%, respectively, were KIR2DL2- and HLA-C1-positive, with 50% presenting the KIR2DL2/HLA-C1 haplotype (Figure 5c, *p* < 0.00001, Chi-square test).

## 4. Discussion

In this age-matched cohort, we identified a consistent immunogenetic and virological signature associated with SARS-CoV-2 infection. Specifically, we observed a significantly higher frequency of the inhibitory KIR2DL2 receptor, an enrichment of its ligand HLA-C1, and an over-representation of the KIR2DL2/HLA-C1 inhibitory pairing among SARS-CoV-2-positive subjects (Figure 1). These findings are consistent with a broad body of evidence showing that KIR/HLA interactions shape NK cell education and activation thresholds and thereby influence antiviral immunity, particularly during herpesvirus infection [16,47]. Importantly, emerging genetic association studies have also implicated specific KIR and HLA variants in COVID-19 susceptibility and/or disease course across independent cohorts [36,37,47,48,49]. In this context, our observation of enrichment of an inhibitory KIR2DL2/HLA-C1 profile in SARS-CoV-2-positive subjects supports the hypothesis that a genetically “more inhibitory” NK cell setting may contribute to suboptimal early cytotoxic control and facilitate viral persistence and immune dysregulation. This interpretation is compatible with reports of NK cell anergy/exhaustion and increased inhibitory receptor dominance in severe COVID-19 [13] and suggests that host KIR/HLA background may be one predisposing factor in the NK cell dysfunction phenotype observed during SARS-CoV-2 infection.

Sex distribution differed between groups, with a higher proportion of females in the SARS-CoV-2-negative group (Table 1). This imbalance likely reflects the well-documented association between male sex and increased risk of severe COVID-19, including higher rates of hospitalization, ICU admission, and mortality [50,51,52,53,54]. Consequently, hospital-based cohorts tend to be high in male patients among SARS-CoV-2-positive and clinically severe cases, even when population-level infection rates are comparable between sexes. Although KIR/HLA genotypes are genetically determined and not influenced by infection status, sex-related differences in antiviral immunity and NK cell function [55,56] could modulate the functional impact of inhibitory KIR2DL2/HLA-C1 interactions. Future studies using sex-balanced cohorts or analyses adjusted for sex will be important to validate these findings and to explore potential sex-specific immunogenetic effects. Although the overall proportion of samples with HHV-6 DNAemia in plasma above 10^2^ copies/mL did not differ between SARS-CoV-2-positive and -negative subjects (Figure 2a), HHV-6 typing revealed a striking divergence (Figure 2b). HHV-6A DNAemia was significantly over-represented in SARS-CoV-2-positive subjects, whereas HHV-6B predominated in SARS-CoV-2-negative subjects. This pattern is biologically plausible because HHV-6A and HHV-6B differ in tropism [57] and immunomodulatory capacity, and both are capable of perturbing NK cell antiviral functions [58]. Importantly, within SARS-CoV-2-positive subjects showing HHV-6A DNAemia, KIR2DL2 and HLA-C1 positivity exceeded 50% (Figure 2c), supporting the concept of a shared susceptibility axis that favors SARS-CoV-2 infection together with HHV-6A reactivation.

Our mortality analysis further suggests clinical relevance among SARS-CoV-2-positive subjects, as in-hospital death was associated with HHV-6 DNAemia (Figure 5a,b); in this dataset, all HHV-6–DNAemic deceased subjects were HHV-6A-positive and frequently carried KIR2DL2, HLA-C1, or the combined inhibitory haplotype (Figure 5c). These observations are consistent with the broader literature reporting herpesvirus reactivation in severe COVID-19 and its association with inflammatory burden and adverse outcomes while also underscoring that HHV-6 is less frequently studied than EBV/CMV and may, therefore, be under-recognized in routine panels [59,60].

In addition, our cross-sectional design does not allow us to determine whether the observed HHV-6A DNAemia, in association with the inhibitory KIR2DL2/HLA-C1 profile, should be interpreted primarily as a biomarker of immune dysregulation or as a contributor to pathogenic mechanisms. On the one hand, herpesvirus DNAemia during acute COVID-19 has often been interpreted as a marker of systemic inflammation and immune perturbation in severe disease [59,61]. On the other hand, HHV-6A has recognized immunomodulatory properties and may influence NK cell function and endothelial responses [44,58,62,63,64,65,66], suggesting that reactivation could plausibly amplify ongoing immune dysfunction in a susceptible subset. Overall, based on the available literature, we consider these findings hypothesis-generating, and longitudinal studies integrating viral kinetics and functional immune profiling will be needed to clarify whether HHV-6A DNAemia is mainly a marker of dysregulation, a mechanistic contributor, or both.

Excess inhibitory NK cell signaling mediated by KIR2DL2 and HLA-C1 might contribute to a permissive environment for both SARS-CoV-2 persistence and HHV-6A reactivation. The KIR2DL2–HLA-C1 interaction delivers inhibitory signals that tune NK activation thresholds, and enrichment of this pairing may be biased toward reduced early cytotoxic control of infected cells when robust innate clearance is most needed [48]. SARS-CoV-2 itself can promote NK dysfunction/exhaustion and inhibitory receptor dominance [13], weakening frontline antiviral surveillance and creating conditions that favor reactivation of latent herpesviruses [21,47]. In parallel, HHV-6 can directly modulate NK biology, including intracellular pathways and regulatory programs that shape effector function [62,63], providing a plausible route by which HHV-6A reactivation could further depress antiviral NK responses, amplify dysregulated inflammation, or contribute to endothelial/vascular injury in predisposed hosts. These effects may be compounded by endothelial damage caused by SARS-CoV-2 [67] and by HHV-6A’s tropism for the vascular endothelium [44,64]. Indeed, HHV-6A endothelial infection has been associated with the induction of proinflammatory and anti-angiogenic effects [65] that may exacerbate vascular injury and related complications. Taken together, these observations are consistent with a hypothesis in which (i) an inhibitory KIR2DL2/HLA-C1 background may lower NK activation thresholds and (ii) SARS-CoV-2-associated NK dysfunction may further compromise antiviral surveillance, thereby potentially favoring HHV-6A DNAemia and related complications. This proposed “two-hit” framework is hypothesis-generating and requires confirmation in larger longitudinal cohorts and mechanistic studies (including NK functional assays and experimental models).

These findings support the inclusion of KIR/HLA genotyping in investigations of COVID-19 susceptibility and clinical outcomes, as well as HHV-6 subtype discrimination (A versus B) when HHV-6 DNAemia is identified, since subtype-specific analyses may reveal biologically and clinically relevant differences. Notably, this integrated approach is feasible in routine practice, as both KIR/HLA genotyping and HHV-6 detection and typing in the present study relied on widely available, cost-effective molecular techniques (conventional PCR, real-time PCR, and nested PCR followed by restriction enzyme digestion), rather than specialized high-throughput platforms. Finally, prospective studies with longitudinal sampling should test whether KIR2DL2/HLA-C1 predicts earlier SARS-CoV-2 viral persistence, higher inflammatory trajectories, or increased probability of HHV-6A DNAemia, and whether HHV-6A DNAemia is primarily a biomarker of immune dysregulation versus a contributor to pathogenic mechanisms.

## 5. Limitations

Key limitations of this preliminary study include the observational, single-time-point design (which precludes inference on temporality or causality), the moderate sample size, particularly for mortality sub-analyses, and reliance on plasma DNAemia thresholds, which may miss cell-associated replication. We assessed HHV-6 DNAemia in plasma to capture circulating viral DNA consistent with reactivation. However, since chromosomally integrated HHV-6 (ciHHV-6) can cause persistently high HHV-6 DNA levels without active replication, most evident in whole-blood/PBMC assays, ciHHV-6 cannot be definitively ruled out and should be addressed in future studies.

Importantly, the present study is observational and based on plasma samples collected at a single time point; therefore, the associations we report cannot establish causality or the temporal sequence between SARS-CoV-2 infection and HHV-6A DNAemia. Several non-mutually exclusive scenarios could explain our findings. First, SARS-CoV-2-driven immune dysregulation, including NK cell dysfunction/exhaustion, may facilitate reactivation of latent herpesviruses, with HHV-6A DNAemia emerging as a consequence of acute infection and/or disease severity. Second, pre-existing or early HHV-6A reactivation could exacerbate COVID-19 by further impairing antiviral NK responses and amplifying inflammatory and endothelial injury pathways, potentially worsening the clinical course in predisposed hosts. Finally, the inhibitory KIR2DL2/HLA-C1 background may represent a shared host susceptibility factor that independently favors both SARS-CoV-2 persistence and HHV-6A reactivation, with HHV-6A DNAemia acting as a marker of immune dysregulation rather than a direct driver of pathology. Longitudinal studies with repeated sampling, ideally pre-/early infection and during follow-up, together with mechanistic in vitro models, will be required to disentangle directionality and define whether HHV-6A is a biomarker or contributor to disease mechanisms.

This single-center study with a moderate sample size may have limited generalizability. In addition, all participants were unvaccinated and infected during circulation of the B.1 lineage, which may restrict applicability to current settings characterized by vaccination, reinfections, and emerging variants. Larger, multicenter longitudinal studies are needed to confirm these findings across diverse populations and epidemiological contexts and to assess the consistency of KIR2DL2/HLA-C1 and HHV-6A DNAemia associations across variant waves and vaccination statuses.

## Figures and Tables

**Table 1:** Demographics of enrolled subjects. * Student’s *t*-test; ^§^ Fisher’s exact test.

	SARS-CoV-2-Negative (n = 110)	SARS-CoV-2-Positive (n = 109)	*p* -Value
**Age** **(Average ± SD)**	74.3 ± 4.2	73.9 ± 7.8	*p* = 0.64 *
**Gender (N M:F)**	34:66	54:55	*p* = 0.0025 ^§^

**Table 2:** Prevalence of clinical comorbidities of enrolled SARS-CoV-2-positive subjects.

Comorbidities	SARS-CoV-2-Positive (N = 109)
**Cardiovascular**	N: 60 (55%)
**Disorders**	
***Hypertension***	*N: 31 (51%)*
***Ischemic colitis***	*N: 9 (16%)*
***Cardiopathy***	*N: 7 (12%)*
***Ictus***	*N: 8 (13%)*
**Chronic Obstructive Pulmonary Disease (COPD)**	N: 20 (18%)
**Diabetes**	N: 23 (21%)
**Mortality**	N: 18 (17%)

## Data Availability

The original contributions presented in this study are included in the article. Further inquiries can be directed to the corresponding authors.

## Abbreviations

The following abbreviations are used in this manuscript:

ACE2	Angiotensin-converting enzyme 2
ARDS	Acute respiratory distress syndrome
ciHHV-6	Chromosomally integrated HHV-6
COPD	Chronic obstructive pulmonary disease
HHV-6	Human herpesvirus 6
HLA-I	Human leukocyte antigen class I
IFN-γ	Interferon-γ
KIR	Killer immunoglobulin-like receptor
ME/CFS	Myalgic encephalomyelitis/chronic fatigue syndrome
NK	Natural killer
SARS-CoV-2	Severe acute respiratory syndrome coronavirus 2
